# Comparison of Respiratory Microbiomes in Influenza Versus Other Respiratory Infections: Systematic Review and Analysis

**DOI:** 10.3390/ijms26020778

**Published:** 2025-01-17

**Authors:** Yunrui Hao, Ying-Jou Lee, Kihan Yap, Miny Samuel, Vincent T. Chow

**Affiliations:** 1 Yong Loo Lin School of Medicine, National University of Singapore, Singapore 117597, Singapore; e1080789@u.nus.edu (Y.H.); e1077821@u.nus.edu (Y.-J.L.); yapkihan@u.nus.edu (K.Y.); medminy@nus.edu.sg (M.S.); 2 Infectious Diseases Translational Research Program, Department of Microbiology and Immunology, Yong Loo Lin School of Medicine, National University of Singapore, Singapore 117545, Singapore

**Keywords:** nasal microbiome, respiratory microbiome, influenza, severity, pediatric patients, adult patients, respiratory infections, pneumonia, COVID-19

## Abstract

Studies have indicated the potential importance of the human nasal and respiratory microbiomes in health and disease. However, the roles of these microbiomes in the pathogenesis of influenza and its complications are not fully understood. Therefore, the objective of this systematic review and analysis is to identify the patterns of nasal and respiratory microbiome dysbiosis and to define the unique signature bacteria associated with influenza compared with other respiratory tract infections. We compared the respiratory microbiome composition between influenza patients and healthy controls; across different influenza severities; in adult versus pediatric influenza patients; as well as influenza versus other respiratory infections. The desired outcomes include the signature bacteria in each cohort and the Shannon index to reflect the alpha diversity. Of the 2269 articles identified, 31 studies fulfilled the inclusion criteria. These studies investigated the respiratory tract microbiomes of patients with influenza, COVID-19, pneumonia, other respiratory infections, and chronic rhinosinusitis (CRS). Our review revealed that the phylum *Firmicutes* and *Actinobacteria*, genus *Actinomyces*, *Streptococcus* and *Granulicatella*, and species *Neisseria* are more prominent in severe influenza than mild to moderate influenza. Reduced microbiome alpha diversity is noted in influenza patients compared to healthy controls. There are some similarities and differences between the signature bacteria in pediatric and adult influenza patients, e.g., *Streptococcus* is common in both age groups, whereas *Pseudomonas* is associated with adults. Intriguingly, there is a common predominance of *Streptococcus* and *Firmicutes* among influenza and pneumonia patients. COVID-19 patients exhibit an increased abundance of *Firmicutes* as well as *Pseudomonas*. In CRS patients, *Proteobacteria* and *Haemophilus* are found in high abundance. This review highlights some similarities and differences in the respiratory microbiomes and their signature organisms in influenza of varying severity and in different age groups compared with other respiratory infections. The dysbiosis of the respiratory microbiomes in these respiratory infections enhances our understanding of their underlying pathogenic mechanisms.

## 1. Introduction

The human upper respiratory tract carries out multiple vital physiologic functions involving the filtration, humidification, and warming of inhaled air while serving as a communication to the lower respiratory and gastrointestinal tracts [1]. The mucosal surfaces of the respiratory tract are colonized by multiple microbial species. The healthy adult nasal microbiome is mainly composed of the five phyla, i.e., *Firmicutes*, *Bacteroidetes*, *Actinobacteria*, *Fusobacteria*, and *Proteobacteria* [2,3,4], while the anterior nares are predominantly colonized by *Actinobacteria* and *Firmicutes* [5]. Other than bacteria, the respiratory tract is also colonized by viruses and fungi.

The variations and types of microbes found on these respiratory mucosal surfaces are already influenced by factors even at birth; the mode of delivery, feeding type, and genetics may vary the composition of the nasal microbiome in infants [6,7]. Later in life, environmental factors such as air humidity, air quality, oxygen, and other factors, including nutrients, smoking, vaccinations, and antibiotic use, also play key roles in shaping the nasal microbiome [8].

Although the roles of the human nasal microbiome in human health have not been fully elucidated, research has indicated the potential importance and association of the nasal microbiota in the pathogenesis of certain conditions [7]. Hence, the nasal microbiome has complex interactions with human hosts, which has implications for health and disease. Dysbiosis of the nasal microbiome has been associated with detrimental effects, e.g., disruption of the homeostasis of the host immune response, thus increasing host susceptibility to respiratory tract infections or RTIs [9]. RTIs may also alter the nasal microbiome, thus indicating the bi-directional nature of this interaction [4]. Dysbiosis of the human respiratory microbiome has been observed in numerous respiratory conditions, such as asthma, chronic rhinosinusitis, bronchiolitis, and COVID-19. Changes in the composition and diversity of the nasal microbiome have been observed in these patients compared to healthy controls, thus suggesting the potential significance of the nasal microbiome in human health and disease [10,11,12,13].

Belonging to the *Orthomyxoviridae* family of RNA viruses, the influenza virus is a contagious pathogen that can cause respiratory disease with a profound impact on human health [14]. Influenza is an extremely prevalent and significant infection associated with seasonal epidemics and pandemics. The World Health Organization estimates about 4 million cases of severe infection and half a million deaths from influenza annually [15]. Clinically, influenza presents with non-specific symptoms, including fever, chills, headache, myalgia, and malaise, accompanied by upper respiratory tract symptoms. Seasonal influenza may be complicated by lower respiratory tract infections (e.g., secondary bacterial pneumonia), as well as systemic complications and manifestations that may be severe [16].

The roles of the nasal microbiome in the pathogenesis of influenza are not well understood. Several studies have explored the differences in the nasal microbiota of healthy controls versus patients. However, these findings are not generalizable due to factors such as the relatively small patient cohorts of observational studies, geographic and regional variations in nasal microbiota, and differences in sampling methods and techniques. Therefore, this study aims to systematically review the literature to identify relevant respiratory microbiome patterns in patients with influenza versus other respiratory infections across various demographic groups. The major objective is to identify patterns of nasal microbiome dysbiosis and to define the bacterial species associated with the various RTIs. A better understanding of the altered composition of human nasal microbiota may potentially improve the management of the pathobionts and reduce infections due to these pathobionts.

The research questions and objectives of this study are as follows:

(a) To compare the differences in human nasal microbiome composition and species between subjects with and without influenza;
(b) To evaluate changes in the human nasal microbiome composition and species as a predictor of influenza severity;
(c) To evaluate the longitudinal changes in the human nasal microbiome in patients with influenza;
(d) To compare differences in the nasal microbiome composition and species in children versus adults with influenza;
(e) To compare differences in the human nasal microbiome in influenza compared to other respiratory infections (such as COVID-19, pneumonia, and chronic rhinosinusitis).

## 2. Materials and Methods

### 2.1. Protocol, Registration and Search Strategies

**Figure 1:**
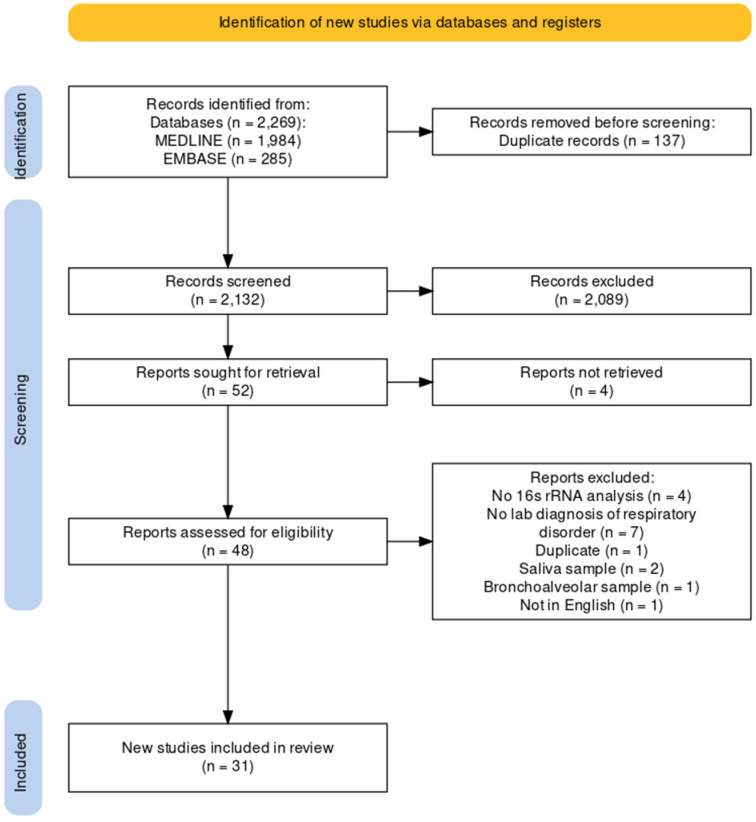
PRISMA flow chart.

We conducted the systematic review according to the PRISMA guidelines (Figure 1). This systematic review was registered in the PROSPERO register (CRD42023408348). The electronic MEDLINE/PubMed and Embase databases were searched for published articles from the inception of each database until 1 January 2023. To achieve the maximum sensitivity of the search strategies, we used combinations of free text and medical subject heading terms, using keywords related to “microbiome”, “influenza”, and “upper airway”. Relevant references, including reviews, were searched for additional studies for consideration.

### 2.2. Study Selection Process and Criteria

The titles and abstracts of studies identified from the electronic databases were first screened by three independent researchers (Y.-J.L., Y.H., and K.Y.) to determine eligibility according to the inclusion and exclusion criteria. Subsequently, full texts of shortlisted studies were obtained and reviewed by three independent researchers (Y.-J.L., Y.H., and K.Y.) to select the final list of studies used in the analysis. If there was a disagreement about the inclusion of a particular study, it was discussed within the whole group to arrive at a consensus. Articles were included in the review if they (a) had study populations of adults or children with laboratory diagnosis of influenza or other respiratory infections, (b) collected samples from nasopharyngeal (NP) or oropharyngeal (OP) or throat swabs or aspirates, (c) targeted 16S rRNA amplicons in their microbiome analysis, (d) were observational cohort studies (including retrospective, prospective, registry, and surveillance designs), randomized controlled trials, case-control studies, or case series, and (e) were published in peer-review journals in English, or with accompanying English translation. To prevent the duplication of patient datasets analyzed, studies originating from the same center(s) during the same or overlapping time periods were collated, and only the most relevant (typically, most recent) study was included for analysis.

Articles were excluded if they: (a) had subjects without laboratory diagnosis of respiratory disorders, (b) had subjects with respiratory tract coinfections other than influenza, COVID-19, pneumonia, and chronic sinusitis (if the population is mixed, papers were excluded only if influenza was not separately analyzed), (c) collected the sample from nasal biopsy, saliva, sputum, or stool samples, and (d) were review articles, commentaries, editorials, letters and correspondences not containing original data, animal studies, interventional studies, case reports, or systematic reviews (including meta-analysis).

### 2.3. Data Collection and Analysis

In the selected articles, data on study group characteristics (number of influenza patients, number and type of comparator/control patients, age, gender, ethnicity, influenza severity, and geographic location), study design and methodology (e.g. sample type), 16S rRNA amplicon(s) targeted, sequencing platform used, and study findings (alpha diversity, beta diversity, and outcome data) were collected.

The data collected included the following:

(a) Nasal microbiome composition and species in influenza versus healthy subjects;
(b) The relationship between nasal microbiome and influenza severity:
  (i) Signature bacterial markers in severe influenza;
  (ii) Microbiome diversity across severe influenza and mild to moderate influenza;
(c) Nasal microbiome composition in adult versus pediatric influenza patients;
(d) Nasal microbiome composition and species in influenza versus other respiratory infections (COVID-19, pneumonia, multiple respiratory infections, or chronic rhinosinusitis).

The results of this systematic literature review and analysis were summarized qualitatively and quantitatively as appropriate.

## 3. Results

### 3.1. Search Results and Selection of Studies

A total of 2269 records were identified from the electronic databases, i.e., MEDLINE (*n* = 1984) and Embase (*n* = 285). After removing duplicates and reviewing titles and abstracts, 48 full reports were assessed for eligibility. Of these, 17 records were excluded for the following reasons: no 16S rRNA analysis, no laboratory diagnosis of the respiratory disorders, duplicate, used saliva and other samples for analysis, or not in English. In the end, 31 studies were included in the review. Details of the inclusion and exclusion of records are provided in Figure 1.

### 3.2. Characteristics of Selected Studies

Supplementary Table S1 shows the characteristics of included studies that assessed the following respiratory infections: influenza, COVID-19, pneumonia, multiple respiratory infections, or chronic rhinosinusitis. All the studies reported results based on respiratory tract microbiomes, i.e., nasopharyngeal microbiome (*n* = 17), oropharyngeal microbiome (*n* = 4), both nasopharyngeal and oropharyngeal microbiome (*n* = 8), and pulmonary microbiome (*n* = 2). These were mainly based on nasopharyngeal swabs (*n* = 17), oropharyngeal swabs (*n* = 4), or both (*n* = 8) and other samples (*n* = 2).

**Figure 2:**
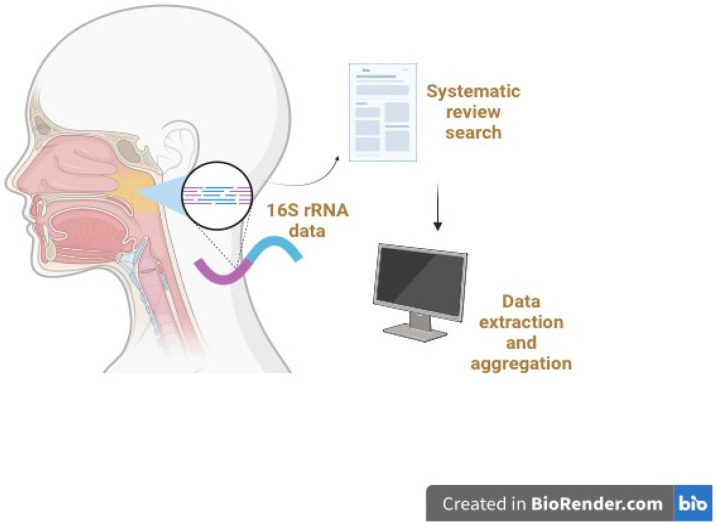
Overview of the 16S rRNA gene sequencing process employed in the selected microbiome studies for systematic review and analysis. Typically, DNA is extracted from the collected nasopharyngeal and other samples and subjected to 16S rRNA gene PCR amplification and library preparation. High-throughput sequencing of the amplified 16S rRNA gene fragments is then performed. Bioinformatics analysis is conducted to analyze the resultant 16S rRNA data to facilitate microbial identification. The systematic review and literature search involve data extraction, aggregation, and detailed analyses.

In terms of genetic analysis, all studies amplified and sequenced 16S ribosomal RNA (rRNA) genes with a variety of selected variable regions, including V1–V2, V1–V3, V3, V4, and V3–V4. Some studies did not specify the variable region. The subject population sizes, clinical status of patients, and signature bacteria are described in Supplementary Table S1. Amongst all studies, signature bacteria were the most commonly reported outcome, followed by the Shannon index as one of the alpha diversity indices. However, only a minority of studies reported the quantitative Shannon index in the main article or Supplementary Materials. Most studies only reported a qualitative “increase” or “decrease” in alpha diversity indices. Figure 2 illustrates the typical 16S rRNA gene sequencing methodology employed in these studies selected for systematic review.

### 3.3. Differences in Human Nasal Microbiomes Between Individuals with Influenza Versus Without Influenza

Supplementary Table S2 provides a qualitative comparison of the nasal microbiomes in subjects with and without influenza. Nine studies were included in the systematic review, whose participants included 825 healthy controls, 474 with influenza, and 129 with other infections. The ages of participants varied from <1 to 68 years. Most of the reports studied the influenza A virus (of unspecified strains), while the others studied the H7N9 virus, influenza B virus, or influenza virus in general. In all nine studies, 16S rRNA gene sequencing was used to analyze the microbiome composition of the nasopharynx or oropharynx of the subjects by amplifying different variable regions (V1–V3, V3–V4, or V4).

At the phylum level, two studies reported a higher abundance of *Firmicutes* and *Actinobacteria* in subjects with influenza infection, whereas a higher abundance of *Bacteroidetes* and *Proteobacteria* was found in the control population [17,18].

At the genus level, pathogenic *Pseudomonas* and *Streptococcus* were found as the signature bacteria in influenza patients across four studies [17,18,19,20], while *Prevotella* was found to be the signature in two studies [21,22].

Additionally, two studies noted that the abundance of Gram-positive commensal species (e.g., *Corynebacterium* and *Lactococcus*) was reduced in influenza patients.

Moreover, there were some inconsistent findings with *Haemophilus*, a pathogenic bacterium. Two studies in China found a higher abundance of *Haemophilus* in healthy control subjects (<16 years old) and in older patients aged 50 to 68 years, respectively [17,18]. However, two other studies in the USA and South Korea found that a higher *Haemophilus* abundance was associated with influenza infection in pediatric patients (<2 years old) and patients aged 0 to 90 years [19,23] (Table 1).

Interestingly, a higher abundance of *Neisseria* was also associated with healthy controls in one study in China [17] but was instead associated with greater influenza severity among patients in another study [24].

From the perspective of nasal microbiome diversity, findings across the four studies suggest that there is a reduced alpha diversity in the influenza-infected population compared to healthy controls [17,21,23,25]. When measured post-infection, the Shannon diversity was reduced in the influenza group, suggesting a change in the uniformity and diversity within the microbiome community. This result is buttressed by significant differences in beta diversity between healthy controls and influenza patients [17,19,20,21], also highlighting the difference in microbiome composition between these two groups.

### 3.4. Changes in the Human Nasal Microbiome Composition and Species of Bacteria in Relation to Influenza Severity

Another aim of our review is to study the association between upper respiratory tract microbiome diversity and influenza severity, as well as to establish signature bacterial species that may reflect influenza severity. Hence, severity was classified into mild-moderate versus severe influenza. Mild-moderate influenza includes classical manifestations such as fever, cough, and runny nose without complications, whereas severe influenza includes classical features together with secondary complications such as secondary bacterial lung infection and neurologic complications. Two other studies defined severity differently; one study compared recovered severe influenza cases versus deceased cases where the fatal outcome was considered severe [18]. Another study defined severe influenza in four parameters, i.e., earlier signs of infection, longer symptom duration, longer viral shedding, and shorter serial interval [24].

As shown in Supplementary Table S2, most studies were on mild-moderate influenza patients, while the others were on either severe or mixed-severity influenza patients. The age range of mild-moderate influenza patients was 2 months to 51 years old, while severe influenza patients ranged from 15 months to 68 years old. Influenza A virus strains were the causative agents in most of the studies, with one study focusing on patients infected with H7N9 avian influenza. In all these studies, 16S rRNA gene sequencing was used to analyze the microbiome composition of the nasopharynx or oropharynx of the subjects by amplifying different variable regions (including V1–V3, V3–V4, and V4).

In terms of microbiome diversity, three studies measured the alpha diversity in patients with different influenza severity and documented increased alpha diversity in cases with more severe influenza. None measured beta diversity across different severities, but only measured between healthy controls and influenza patients (Supplementary Table S2).

While there is no significant association between alpha diversity prior to infection and symptom duration, Lee et al. [24] reported that individuals with higher alpha diversity displayed longer viral shedding and shorter serial interval (defined as the time between symptom onset of an index case and a secondary case, as indicated by the Shannon and Chao index). This study also reported that the time to shedding onset was significantly reduced in patients with a more enriched nasal microbiome diversity. These findings align with the study by Langevin et al. [19], which found that greater influenza severity was associated with increased bacterial alpha diversity in the upper respiratory tract, as indicated by the Shannon and Chao index (Table 1).

Furthermore, some studies reported that increased alpha diversity in influenza A patients was associated with a greater incidence of serious secondary complications, including secondary bacterial lung infection (SBLI) and severe neurologic complications [18,19].

Patients with severe influenza harbored a significantly greater abundance of bacteria such as *Firmicutes*, *Actinobacteria*, *Actinomyces*, and *Streptococcus* compared to mild-moderately infected patients or healthy controls [9,17,18,19,26].

Aside from the more common bacterial biomarkers, other studies observed a greater abundance of *Prevotella* and *Veillonella* genera in severe influenza [17,19]. One study reported a greater abundance of *Staphylococcus aureus* in milder influenza [19].

In addition, another study found that the *Neisseria* oligotype was associated with shorter serial intervals, early signs of infection, longer symptom duration, and viral shedding, thus alluding to its possible association with severe influenza [23].

Supplementary Table S2 summarizes the signature bacterial species linked to different influenza severity across various studies and outlines each study’s method of assessing influenza severity.

### 3.5. Longitudinal Changes in the Human Nasal Microbiome in Patients with Influenza

Only one study investigated the longitudinal changes in nasal microbiomes in patients with influenza [22] but found no significant changes in Shannon index and beta diversity with duration of infection. However, this study found an altered abundance of operational taxonomic units (OTUs) from 3 to 6 days post-infection (dpi). On 3 dpi, *Prevotella melaninogenica*, *Leptotrichia*, human oral taxon 352, and *Porphyromonas* were more abundant in influenza cases. On 6 dpi, *Fusobacterium necrophorum* and *Prevotella* were more abundant in influenza patients.

At the phylum level, there were changes in abundance over the period of influenza infection. The abundance of *Actinobacteria* significantly increased by 6 dpi compared to baseline, which then returned to baseline level by 28 dpi.

At the genus level, influenza patients exhibited a significant increase in *Prevotella* abundance at 3 and 28 dpi, *Actinomyces* abundance at 6 dpi, and *Fusobacterium* abundance at 28 dpi. However, *Haemophilus* abundance significantly decreased.

Overall, this study showed alterations in the abundance of different bacterial species in the nasal microbiome throughout the course of influenza infection.

### 3.6. Differences Between Nasal Microbiomes of Children and Adults with Influenza

Table 2 summarizes the 13 studies that characterized the nasal microbiomes of adult patients and pediatric patients with influenza. The ages of the participants varied from children aged 0 to 16 years old to adults aged 68 years old. Most of the studies recruited patients infected with influenza A virus strains, including H1N1 and H7N9. In all studies, 16S rRNA gene sequencing analyzed the microbiome composition of the nasopharynx or oropharynx of the subjects by amplifying different variable regions (including V1–V3, V3–V4, and V4).

In pediatric patients, there were inconsistent results with regard to the effect of influenza on the richness and evenness of the nasal microbiome composition. Of the three pediatric studies, one study suggested an increase in alpha diversity in cases compared to controls [21], one study suggested a decrease in alpha diversity in cases [17], and the other did not compare the alpha diversity in cases versus controls [19].

In contrast, the findings in the ten adult influenza studies were more consistent, with four studies reporting a decline in alpha diversity in cases versus controls [9,20,23,25]. However, one study reported an increase in alpha diversity in cases versus controls [18], two reported insignificant findings [22,27], and three did not compare alpha diversity between cases and controls [24,26,28].

With regard to beta diversity in three pediatric studies, two studies reported a significant difference in beta diversity between cases and controls [17,21], while one study did not calculate beta diversity [19]. For adult influenza, six studies reported a significant difference in beta diversity between cases and controls [9,18,20,24,25,27], one study reported insignificant findings [22], and three studies did not determine beta diversity [23,26,28].

At the phylum level, only one study reported an increase in the abundance of *Firmicutes* and *Actinobacteria* in pediatric patients [17]. Adults with influenza still have the same five predominant phyla as healthy controls, i.e., *Bacteroidetes*, *Proteobacteria*, *Firmicutes*, *Fusobacteria*, and *Actinobacteria*. However, adults with influenza exhibit an increased abundance of *Firmicutes*, *Actinobacteria*, and *Fusobacteria*, which is similar to pediatric influenza patients (except for an additional increase in abundance of *Fusobacteria*) [18].

Although two studies on influenza patients reported *Streptococcus* as the main genus that increased in abundance, one study found a reduction in *Streptococcus* [21]. In adults, *Pseudomonas* appeared to be the signature genus associated with influenza in adult patients, as reported by three studies [18,20,28]. Additionally, one study also reported *Streptococcus* as the signature genus [27]. Interestingly, *Prevotella* has been reported as the signature microorganism in two adult studies and one pediatric study.

**Table 2:** Comparison of signature microorganisms associated with nasal microbiomes of pediatric versus adult patients with influenza and other respiratory infections.

Study Subjects	Sample	Gene Sequencing of Variable Region	Signature Microorganisms Associated with Influenza (or Other Respiratory Infections)	References
**Pediatric patients**				
IAV patients, Healthy controls	OP swab	16S rRNA V3–V4	*Streptococcus* *Actinomyces* *Lactobacillales* *Veillonellaceae*	Hu et al., 2022 [17]
IAV patients	NP swab	16S rRNA	*Streptococcus* *Moraxella* *Staphylococcus* *Haemophilus*	Langevin et al., 2017 [19]
Influenza patients, MP patients, Healthy controls	NP swab OP swab	16S rRNA V3–V4	*Prevotella*	Zhou et al., 2020 [21]
**Adult patients**				
IAV patients, Healthy controls, Other common virus infections	NP swab	16S rRNA V1–V3	*Pseudomonas*	Kaul et al., 2020 [20]
IAV patients, Healthy controls	NP swab OP swab	16S rRNA V4	*Neisseria*	Lee et al., 2019 [24]
IAV H7N9 patients, Healthy controls	OP swab Nasal lavage	16S rRNA	*Pseudomonadaceae* *Fusobacteria* *Bifidobacteriaceae* *Bacteroidaceae*	Lu et al., 2017 [18]
IAV patients, Healthy controls	OP swab	16S rRNA V1–V3	*Prevotella*	Ramos-Sevillano et al., 2019 [22]
IAV and IBV patients, COVID-19 patients	NP swab	16S rRNA V4	*Enterobacteriaceae*	Rattanaburi et al., 2022 [25]
IAV and IBV patients	NP swab OP swab	16S rRNA V4	*Prevotella*	Tsang et al., 2020 [26]
IAV with severe acute respiratory infection	NP aspirate	16S rRNA V4	*Streptococcus*	Borges et al., 2018 [27]
Non-IAV with severe acute respiratory infection				
IAV H1N1 patients	Endotracheal aspirate BAL	16S rRNA V3–V4	*Proteobacteria* *Bacteroidetes* *Firmicutes*	Hernández-Terán et al., 2023 [9]
IAV H7N9 patients, Healthy controls	OP swab	16S rRNA V3–V4	*Pseudomonas*	Zha et al., 2020 [28]
Influenza, parainfluenza, rhinovirus, RSV, COVID-19, adenovirus, metapneumovirus patients, Healthy controls	NP aspirate Sputum OP swab	16S rRNA V1–V3	*Haemophilus* *Moraxella*	Yi et al., 2014 [23]

IAV: Influenza A virus; IBV: Influenza B virus; MP: *Mycoplasma pneumoniae*; RSV: Respiratory syncytial virus; NP: Nasopharyngeal; OP: Oropharyngeal; BAL: Bronchoalveolar lavage.

### 3.7. Differences in the Human Nasal Microbiome in Influenza Compared to Other Respiratory Infections and Conditions (Including COVID-19)

Table 3 illustrates nine studies that characterized the nasal microbiomes of subjects with respiratory infections or inflammatory conditions other than influenza. Five studies focused on pneumonia, two on COVID-19, and two on chronic rhinosinusitis (CRS). Out of the nine studies, only two directly compared influenza against other respiratory infections. In all nine studies, 16S rRNA gene sequencing was used to analyze the microbiome composition of the nasopharynx or oropharynx of the subjects by amplifying different variable regions (V1–V2, V1–V3, V3–V4, V3, and V4).

Two studies reported a decrease in alpha diversity in pneumonia patients versus controls [21,29]. Three other pneumonia studies [30,31,32] and both studies on COVID-19 [25,33] did not report alpha diversity against controls. Both studies on CRS reported reduced alpha diversity in cases versus controls [34,35]. One study reported a significant decrease in alpha diversity in influenza A versus COVID-19 patients (*p* < 0.05) [25].

Only one study on pneumonia patients reported a significant difference in beta diversity in cases versus controls (*p* = 0.001) [21]. Significant differences in beta diversity between cases versus controls were also found in one study on COVID-19 (*p* < 0.05) [25] and in one study on CRS patients (*p* < 0.01) [34].

With respect to nasal microbiome composition, the taxonomic composition differed between influenza and other respiratory infections, albeit with some commonalities. As stated previously, influenza patients have *Actinobacteria*, *Firmicutes*, *Pseudomonas*, and *Streptococcus* predominating in their nasal microbiomes. In studies on pneumonia patients, multiple common signature bacteria were found to be predominating, i.e., *Streptococcus* (*n* = 4), *Staphylococcus* (*n* = 3), *Firmicutes* (*n* = 2), and *Mycoplasma* (*n* = 2). Intriguingly, there is a common predominance of *Streptococcus* and *Firmicutes* among influenza and pneumonia patients.

Both studies on COVID-19 found an increased abundance of *Firmicutes* in COVID-19 patients (mainly infected with the original SARS-CoV-2), in common with influenza infection. Moreover, one study found an increase in *Pseudomonas*, which is also common in influenza infection.

In both studies on CRS patients, *Proteobacteria* and *Haemophilus* were found in high abundance, which was somewhat different from influenza patients.

**Table 3:** Comparison of signature microorganisms associated with nasal microbiomes of patients with respiratory infections and conditions other than influenza.

Study Subjects	Sample	Sequencing Method	Signature Bacteria	References
**Pneumonia**				
*Mycoplasma pneumoniae* pneumonia	NP swab OP swab BAL (additional in sick children)	16S rRNA V3–V4	*Staphylococcus* *Corynebacterium* *Mycoplasma*	Dai et al., 2018 [30]
Healthy controls				
Pneumonia	NP swab	16S rRNA V3	*Haemophilus* *Staphylococcus* *Streptococcus*	Kelly et al., 2017 [31]
Healthy controls				
Pneumonia	NP swab	16S rRNA V1–V2	Viral cause: *Moraxella lacunata* Non-viral cause: *Streptococcus pneumoniae*, *Haemophilus influenzae*, *Moraxella catarrhalis*	Sakwinska et al., 2014 [29]
Healthy controls				
Community-acquired pneumonia	NP swab OP swab	16S rRNA V1–V3	*Actinobacteria* (*Actinomyces*), *Firmicutes* (*Streptococcus pneumoniae*, *Staphylococci*)	Weimken et al., 2015 [32]
*Mycoplasma pneumoniae* pneumonia	OP swab Nasal lavage	16S rRNA	Phylum: *Firmicutes* Genus: *Mycoplasma*, *Lactobacillus*, *Ralstonia*, *Acinetobacter*, *Actinomyces* NP only: *Streptococcus* OP only: *Corynebacterium*	Zhou et al., 2020 [21]
Influenza				
Healthy controls				
**COVID-19**				
IAV and IBV	NP swab	16S rRNA V4	Phylum: *Firmicutes*, *Bacteroidetes* Genus: *Enterobacteriaceae*, *Staphylococcus*, *Lautropia*, *Pseudomonas*, *Corynebacterium*	Rattanaburi et al., 2022 [25]
COVID-19				
COVID-19-positive respiratory infection	NP swab	16S rRNA V3–V4	Phylum: *Proteobacteria*, *Firmicutes*, *Actinobacteria*	Tchoupou Saha et al., 2022 [33]
COVID-19 negative respiratory infection				
**Chronic Rhinosinusitis**				
Non-asthmatic CRSwNP patients	Nasal swab	16S rRNA V1–V2	Phylum: *Proteobacteria* Genus: *Haemophilus* Species: *Haemophilus influenzae*, *Corynebacterium pseudodiphtheriticum*	Chalermwatanachai et al., 2018 [34]
CRSwNP patients with asthma				
Healthy controls				
CRSsNP CRSwNP	Anterior nares, NP, maxillary, and ethmoid sinus swabs (patients) Anterior nares, NP swab (controls)	16S rRNA V4	*Staphylococcus* *Corynebacterium* *Moraxella* *Haemophilus* *Streptococcus* *Prevotella*	De Boeck et al., 2019 [35]
Healthy controls				

CRSsNP: Chronic rhinosinusitis without nasal polyps; CRSwNP: Chronic rhinosinusitis with nasal polyps; NP: Nasopharyngeal; OP: Oropharyngeal; BAL: Bronchoalveolar lavage.

## 4. Discussion and Conclusions

**Figure 3:**
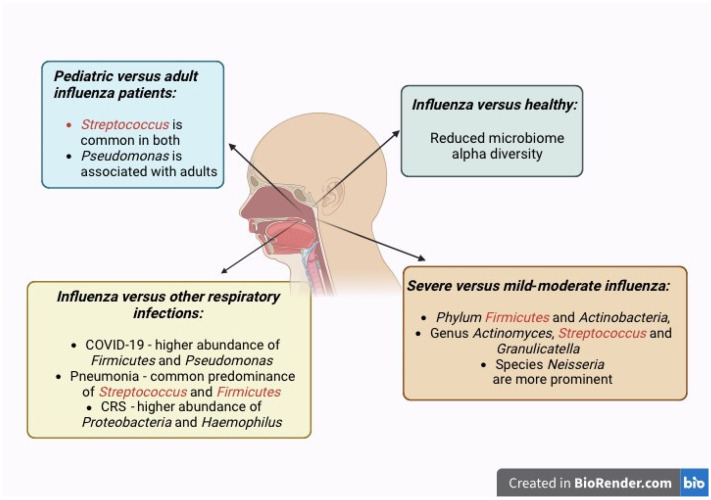
The nasopharyngeal microbiome is modulated by influenza virus infection, which alters the healthy microbiome with commensal microbiota into the pathobiome with potentially pathogenic microbiota. These changes in microbial communities culminate in microbial dysbiosis. Representation of the increased abundance of specific organisms in the nasal microbiome of influenza infection in relation to disease severity, pediatric versus adult patients, and in comparison to other respiratory infections and conditions.

Our analyses of respiratory microbiome differences between influenza patients and healthy controls in multiple studies suggest that RTIs cause dysbiosis of the nasal microbiome, as reflected by changes in the microbiome composition, as well as significantly reduced beta diversity in influenza patients (Figure 3). This finding is also commonly shared among the adult and pediatric populations, indicating that RTIs cause dysbiosis in both age groups. However, the alpha diversity indices differ between these two populations. This is in agreement with the dissimilar composition and abundance of organisms found in the two populations. The different microbiome alterations between adult and pediatric patients may be attributed to the different baseline composition and alpha diversity of their nasal microbiomes [8].

Detailed examination of individual components of the microbiome revealed a higher dominance of pathogenic organisms (i.e., *Pseudomonas* and *Streptococcus*) in patients compared to healthy controls across the studies. Interestingly, *Pseudomonas* is also more abundant in other respiratory pathologic conditions, including cystic fibrosis, chronic obstructive pulmonary disease (COPD), and bronchiectasis [36,37]. This suggests that certain bacterial species may be common in dysbiosis of nasal microbiomes in certain respiratory pathologies.

However, due to the inconsistent time of sample collection in different studies, it is difficult to establish a distinct temporal relationship between influenza infection and nasal microbiome dysbiosis. For instance, *Streptococcus* is associated with a less stable microbiome, which may further exacerbate dysbiosis and compromise the host immune response [38]. *Streptococcus* abundance is also associated with the upregulation of adhesion receptors for viral entry [39]. Despite the association between *Pseudomonas*, *Streptococcus*, and influenza infection, the cause–effect relationship remains unclear as to whether dysbiosis predisposes to influenza infection or vice versa. Notwithstanding this, there is one study that evaluated the longitudinal alterations in nasal microbiome over time, and more such studies are warranted to address this relationship.

Our review noted a reduction in certain Gram-positive commensal bacteria, namely *Corynebacterium* and *Lactococcus*, in influenza patients. These bacteria can confer protective effects against airway infections by regulating the colonization of pathogenic organisms such as *Streptococcus pneumoniae* and *Haemophilus influenzae*, and thus reduce the risk of complications. Hence, a reduction in these bacteria may exacerbate the severity of influenza, thus rendering them a potential prognostic biomarker [19,40].

Some inconsistencies in the composition of organisms, namely *Haemophilus* and *Neisseria*, may be attributed to the different baseline alpha diversity amongst different demographics, e.g., age groups and geographic and ethnic factors. Interestingly, *Haemophilus* is also a pathogenic bacterium with a similar effect as *Streptococcus*, i.e., by decreasing microbiome stability and upregulating adhesion receptors for viral entry [38,39]. Further studies are necessary to establish an association between *Haemophilus*, *Neisseria*, and influenza infection.

Our review found that *Firmicutes* and *Actinobacteria*, *Actinomyces*, *Streptococcus*, *Granulicatella*, and *Neisseria* are more prominent in severe influenza compared to mild or moderate influenza [9,17,18,19,26]. Interestingly, *Neisseria* species have been identified as pathobionts in bronchiectasis [41].

Influenza virus infection may perturb the normal microbiome composition and culminate in the overgrowth of pathogenic bacteria such as *Actinomyces*, *Streptococcus*, and *Neisseria*. In addition, *Streptococcus* and *Neisseria* may share a symbiotic relationship with influenza virus. Influenza may augment airway transmission of bacteria by enhancing their adherence and colonization of the respiratory epithelium. In turn, bacteria may modify the host immune response to favor viral replication, e.g., by secreting viral hemagglutinin-activating proteases or by acting as mucosal adjuvants to promote influenza virus entry. These mutually beneficial mechanisms may cooperate and result in more severe clinical manifestations such as secondary bacterial pneumonia [42,43,44].

Increased abundance of *Prevotella* and *Veillonella* in certain studies may be explained by their role as anaerobic Gram-negative bacteria, which may cause anaerobic pulmonary infections [19]. In milder influenza, *Staphylococcus aureus* is more abundant, which concurs with previous findings on its preventive effect in influenza-mediated lung injury in murine models [45].

The observation that airway alpha diversity is diminished in influenza patients compared to healthy controls but is higher in more severe influenza than in mild-moderate influenza may appear contradictory. However, the former finding in influenza patients indicates the loss of nasal commensal flora which may offer protection against infections. In contrast, higher alpha diversity in more severe influenza may be due to increased colonization by pathogenic organisms, which disrupts the microbiome equilibrium.

Comparing influenza with other respiratory infections, there are similarities in microbiome alterations. For example, there are similar increases in the abundance of *Firmicutes* in influenza, COVID-19, and pneumonia and of *Streptococcus* in influenza and pneumonia. This suggests certain similarities in perturbations of the otherwise healthy nasal microbiome. Moreover, it also implies that similar clinical applications may be adopted for certain infections, e.g., facilitating early targeted interventions by monitoring, regulating, and even restoring the nasal microbiome to its healthy state.

Although we managed to identify a number of signature bacteria associated with differing influenza severity, this review was limited by the number of studies and variations in the methods and subjects recruited by different studies. For instance, we could only find three studies that investigated nasal microbiomes in pediatric influenza patients. Moreover, some observations regarding *Haemophilus* and *Neisseria* still remain inconsistent in studies reported thus far. This restricted our ability to identify more signature bacteria due to the limited number of studies from which to draw conclusions. This is critical since the different baseline alpha diversity amongst different studies can be attributed to varying lifestyle, geographic, and genetic factors [46]. Indeed, influenza patients may exhibit patterns of signature bacteria that have yet to be discovered or established, which may be missed in the current studies. Furthermore, many studies only determined the nasal microbiome composition at one time-point post-infection, rendering it difficult to (a) draw meaningful conclusions regarding longitudinal changes in microbiota across the entire duration of the infection, and (b) establish a causality behind the observed patterns. The current lack of this temporal relationship makes it challenging to decide whether the observed findings are attributed to influenza or whether dysbiosis predisposes to influenza virus infection.

Therefore, we propose the following study design for future investigations: a prospective study that follows a group of subjects before influenza infection and collects nasal or respiratory samples at multiple time-points immediately after influenza infection right up to the end-point of disease and recovery. This facilitates precise monitoring of the dynamic alterations in the respiratory microbiome throughout the whole course of the infection and the establishment of a well-defined temporal relationship in order to accurately identify the signature bacteria that are distinctly associated with greater influenza susceptibility and higher influenza severity.

More such investigations into the respiratory microbiota are thus necessary in different populations to develop better prognostic tools and targeted interventions to ameliorate the morbidity and mortality associated with influenza and related infections [47,48,49,50].

In conclusion, this review provides a current and systematic analysis of respiratory microbiome differences during influenza virus infection in different demographics and provides an overall view of the common patterns of microbiome alterations and signature bacteria observed. We conclude that *Firmicutes* and *Actinobacteria*, *Actinomyces*, *Streptococcus*, *Granulicatella*, and *Neisseria* are more prominent in severe influenza compared to moderate influenza. Decreased microbiome alpha diversity is noted among influenza patients as compared to healthy controls. Knowledge of the prominent signature organisms in the respiratory microbiome is valuable as these bacteria can serve as potential biomarkers to distinguish between different severities of influenza. By understanding the microbiome dysbiosis observed in more severe influenza, future strategies can be explored to modulate the host respiratory microbiome responses to potentially prevent exacerbation of influenza infection. Ultimately, early targeted clinical interventions may be designed to prevent serious complications that drastically escalate influenza morbidity and even mortality.

## Figures and Tables

**Table 1:** Characteristics of studies on nasal microbiome diversity in patients with different influenza disease severity.

References	Type of Sample	Disease Severity (*n*)	Alpha Diversity (Shannon Index)	Alpha Diversity (Simpson Index)	Alpha Diversity (Chao Index)
Langevin et al., 2017 [19]	NP swab	14 severe influenza	Severe vs. mild: Influenza outcome = 12.44 vs. 0.0014	Severe vs. mild: Influenza outcome = 14.66 vs. 0.0006	Severe vs. mild: Influenza outcome = 10.72 vs. 0.0027
		22 mild influenza	Days since symptom onset = 0.9 vs. 0.3519	Days since symptom onset = 2.49 vs. 0.1258	Days since symptom onset = 0.24 vs. 0.6314
Lee et al., 2019 [24]	NP swab, OP swab	124 index cases	Shedding duration: 25th and 75th quartiles of Shannon index = 3.1 and 3.6 days	-	Serial interval: Chao index AF 0.992; 25th and 75th quartiles of Chao index = 3.8 and 3.0 days
			Serial interval: Shannon index AF 0.72; 25th and 75th quartiles of Shannon index = 3.7 and 3.2 days		Time to shedding onset: AF 0.995; 25th and 75th quartiles of Chao index = 5.8 and 5.2 days
Lu et al., 2017 [18]	OP swab, nasal lavage	21 severe	Graphical representation only		
		30 mild-moderate			

NP: Nasopharyngeal; OP: Oropharyngeal; AF: Acceleration factor.

## Supplementary Materials

The following supporting information can be downloaded at https://www.mdpi.com/article/10.3390/ijms26020778/s1.
