# Supplementary material for: Comparison of Respiratory Microbiomes in Influenza Versus Other Respiratory Infections: Systematic Review and Analysis
Source: Int J Mol Sci. 2025 Jan 17;26(2):778. doi: 10.3390/ijms26020778 (PMC11765715; doi:10.3390/ijms26020778)
Supplement: Supplementary file 1 [file ijms-26-00778-s001.zip › IJMS Microbiome Supp Table S1 16Jan2025.pdf]

Supplementary Table S1. Characteristics of the selected studies on the respiratory microbiomes in healthy controls versus patients with influenza or other respiratory infections and conditions.

References	Country	Study Design	Disease(s)	Age	Type of Sample	Gene Sequencing	Disease (*n*)	Comparison (n)
Yi et al. 2014 [23]	South Korea	Cross-sectional study	Influenza, parainfluenza, rhinovirus, RSV, COVID-19, adenovirus, metapneumovirus	No limit	NP aspirate<br>Sputum<br>OP swab	16S rRNA<br>V1-V3	Respiratory infection = 59	Healthy controls = 57
Langevin et al. 2017 [19]	USA	Cohort study	IAV	<2 years	NP swab	16S rRNA	Mild IAV = 22<br>Severe IAV = 14	-
Lu et al. 2017 [18]	China	Cohort study	IAV H7N9	50 - 68 years	OP swab<br>Nasal lavage	16S rRNA<br>V3-V4	H7N9 = 30<br>H7N9 + SBLI = 21	Healthy controls = 30
Borges et al. 2018 [27]	Brazil	Case-control study	IAV (with severe ARI)	<1 - 60 years	NP aspirate	16S rRNA<br>V4	IAV (with severe ARI) = 6	Non-IAV (with severe ARI) = 6
Lee et al. 2019 [24]	Nicaragua	Cohort study	IAV	No limit	NP swab<br>OP swab	16S rRNA<br>V4	IAV = 144	Household contacts = 573
Ramos-Sevillano et al. 2019 [22]	United Kingdom	Case-control study	IAV	18 - 45 years	OP swab	16S rRNA<br>V1-V3	IAV = 43	Healthy controls = 35
Kaul et al. 2020 [20]	Chile	Cohort study	IAV	No limit	NP swab	16S rRNA<br>V1-V3	H1N1pdm09 / H3N2 = 28	Healthy controls = 22
Tsang et al. 2020 [26]	Nicaragua	Cohort study	IAV, IBV	No limit	NP swab<br>OP swab	16S rRNA<br>V4	Influenza = 115	Household contacts = 436
Zhou et al. 2020 [21]	China	Case-control study	Influenza	Unspecified	NP swab<br>OP swab	16S rRNA<br>V3-V4	Influenza = 66	MPP = 40<br>Healthy controls = 59
Zha et. al. 2020 [28]	China	Cohort study	IAV H7N9	50 - 68 years	OP swab	16S rRNA<br>V3-V4	H7N9 = 21<br>H7N9 + SBLI = 21	Healthy controls = 30
Hu et al. 2022 [17]	China	Cohort study	IAV	<16 years	OP swab	16S rRNA<br>V3-V4	IAV = 49	Healthy controls = 42
Rattanaburi et al. 2022 [25]	Thailand	Cohort study	IAV<br>COVID-19	Unspecified	NP swab	16S rRNA<br>V4	IAV = 24<br>IBV = 24	COVID-19 patients = 24<br>Non-influenza / non-COVID-19 = 24

Hernández-Terán et al. 2023 [9]	Mexico	Case-control study	IAV H1N1pdm09 (deceased vs. recovered patients)	36.5 - 51 years	Endotracheal aspirate<br>Bronchoalveolar lavage	16S rRNA<br>V3-V4	Deceased IAV patients = 5	Recovered IAV patients = 25
			Prior use of antibiotics before hospitalization				IAV + antibiotic use = 23	IAV + non-antibiotic use = 7
Sakwinska et al. 2014 [29]	Switzerland	Case-control study	Pneumonia	2 months - 16 years	NP swab	16S rRNA<br>V1-V2	Pneumonia = 50	Healthy controls = 50
Weimken et al. 2015 [32]	USA	Retrospective data analysis of RCT	Community-acquired pneumonia (unknown etiology)	35 - 91 years	NP swab<br>OP swab	16S rRNA<br>V1-V3	Community-acquired pneumonia = 10	-
Kelly et al. 2017 [31]	Botswana	Cohort study	Pneumonia	Unspecified	NP swab	16S rRNA<br>V3	Pneumonia = 204	Healthy controls = 60<br>URTI = 55
Lu et al. 2017 [18]	USA	Case-control study	Pneumonia	0 - 12.7 years	NP swab<br>OP swab	16S rRNA<br>V3-V4	Pneumonia = 60	Healthy controls = 89
Dai et al. 2018 [30]	China	Cohort study	*Mycoplasma pneumoniae* pneumonia (MPP)	Unspecified	NP swab<br>OP swab<br>BAL (additional in sick children)	16S rRNA<br>V3-V4	MPP = 28	Healthy controls = 32
Tchoupou Saha et al. 2022 [33]	France	Cohort study	COVID-19	No limit	NP swab	16S rRNA<br>V3-V4	COVID-19 = 90	COVID-19 negative = 3
Chalermwatanachai et al. 2018 [34]	Belgium	Cohort study	Chronic rhinosinusitis with nasal polyp (CRSwNP)	No limit	NP swab	16S rRNA<br>V1-V2	CRSwNP (without asthma) = 21<br>CRSwNP (with asthma) = 20	Healthy controls = 17
De Boeck et al. 2019 [35]	USA	Cohort study	Chronic rhinosinusitis (CRS)	Unspecified	Anterior nares, NP swab, maxillary and ethmoid sinus swabs (patients)<br>Anterior nares, NP swab (controls)	16S rRNA<br>V4	CRS = 190	Healthy controls = 100
Toivonen et al. 2019	Finland	Prospective cohort study	Acute respiratory infection (ARI)	<24 months	NP swab	16S rRNA<br>V4	ARI = 839	-
Hoefnagels et al. 2021	Netherlands	Cohort study	Lower respiratory tract infection	1 - 60 months	NP swab	16S rRNA	Single virus = 94<br>2 viruses = 32<br>>2 viruses = 13	-

Mathew et al. 2016	Netherlands	Cohort study	Severe pneumonia (bacterial, viral)	1 - 144 months	NP aspirate	-	Pneumonia = 67	Severe pneumonia (without organism) = 82
Stewart et al. 2017	USA	Prospective cohort study	Bronchiolitis	<12 months	NP aspirate	16S rRNA V4	Bronchiolitis (with positive pressure ventilation use) = 25	Bronchiolitis (without positive pressure ventilation use) = 119
Rosas-Salazar et al. 2022	USA	Cohort study	Respiratory syncytial virus (RSV)	0 - 12 months	Nasal wash	16S rRNA V4	RSV = 357	-
Howard et al. 2016	USA	Prospective cohort study	SPN colonization of nasal microbiome	6 - 12 months	NP wash	16S rRNA V4	SPN colonization = 53	No SPN colonization = 104
Coleman et al. 2021	Australia	Cohort study	Otitis media (OM)	2 - 7 years	Buccal swab<br>Palatine tonsil swab<br>Throat swab	16S rRNA V3-V4	Current OM or history of OM = 86	Healthy controls = 17
Ruohola et al. 2013	Finland	Cohort study	Acute otitis media (AOM)	6 - 35 months	NP sample	Culture, PCR, antigen	AOM = 318	Non-AOM = 187

RCT: Randomized control trial; NP: Nasopharyngeal; OP: Oropharyngeal; IAV: Influenza A virus; IBV: Influenza B virus; SBLI: Secondary bacterial lung infection; SPN: *Streptococcus pneumoniae*; URTI: Upper respiratory tract infection
