# Supplementary material for: Comparison of Respiratory Microbiomes in Influenza Versus Other Respiratory Infections: Systematic Review and Analysis
Source: Int J Mol Sci. 2025 Jan 17;26(2):778. doi: 10.3390/ijms26020778 (PMC11765715; doi:10.3390/ijms26020778)
Supplement: Supplementary file 1 [file ijms-26-00778-s001.zip › IJMS Microbiome Supp Table S2 16Jan2025.pdf]

Supplementary Table S2. Nasal and respiratory microbiome composition and species in healthy controls versus patients with influenza (by disease severity) or other respiratory infections.

References	Study Subjects	Type of Sample	Gene sequence	Signature Species Associated with Influenza	Signature Species Associated with Healthy Subjects	Influenza (*n*)	Comparison (*n*)	Influenza Severity		Severity Criteria	Signature Species Associated with Influenza in Relation to Severity (where available)
								Mild-moderate (*n*)	Severe (*n*)		
**Pediatric Subjects**											
Zhou et al. 2020 [21]	Influenza patients, MP patients, Healthy controls	NP swab, OP swab	16S rRNA V3-V4	**Genus**: *Prevotella*	-	66	MP patients = 40 Healthy controls = 59	66	-	Typical clinical symptoms (fever, cough, dyspnea, vomiting)	**Mild-moderate:** *Prevotella*
Hu et al. 2022 [17]	IAV patients, Healthy controls	OP swab	16S rRNA V3-V4	**Phylum:** *Firmicutes, Actinobacteria* **Genus**: *Actinomyces, Streptococcus, Lactobacillales, Veillonella*	**Phylum**: *Bacteroidota, Proteobacteria, Fusobacteriota* **Genus**: *Haemophilus, Neisseria, Alloprevotella, Leptotrichia*	49	Healthy controls = 42	-	49	IAV pneumonia	**Severe:** *Actinomyces, Streptococcus, Lactobacillales, Veillonella*
Langevin et al. 2017 [19]	IAV patients	NP swab	16S rRNA V1-V3	**Genus**: *Moraxella, Streptococcus, Haemophilus, Staphylococcus*	-	22 mild influenza<br><br>14 severe influenza	-	22	14	**Severe:** Children with complications (need ICU)	**Mild-moderate:** *Staphylococcus aureus*<br><br>**Severe:** *Moraxella, Prevotella, Streptobacillus, Porphyromonas, Veillonella, Fusobacterium, Haemophilus, Lachnospiracea incertae sedi, Granulicatella* *Moraxella catarrhalis, Prevotella melaninogenica, Veillonella dispar, Granulicatella elegans*

**Adult Subjects**											
Yi et al. 2014 [23]	Influenza, parainfluenza, rhinovirus, RSV, coronavirus, adenovirus, metapneumovirus<br><br>Healthy controls	NP aspirate, sputum, OP swab	16S rRNA V1-V3	**Genus**:<br>*Haemophilus*, *Moraxella*	**Genus**:<br>*Streptococcus*, *Neisseria*, *Gemella*, *Aggregatibacter*, *Actinobacillus*	7	Parainfluenza = 24, Rhinovirus = 8, RSV = 14, COVID-19 = 4, Adenovirus = 1, Metapneumovirus = 1<br>Healthy controls = 40	-	-		
Lu et al. 2017 [18]	IAV H7N9 patients, Healthy controls	NP swab, OP swab	16S rRNA V3-V4	**Phylum**:<br>*Actinobacteria*, *Firmicutes*<br>**Genus**:<br>*Pseudomonas*	**Phylum**:<br>*Bacteroidetes*<br>**Genus**:<br>*Haemophilus*	51	Healthy controls = 30	30	21	**Mild-moderate:**<br>Symptomatic without complications<br>**Severe:**<br>Secondary bacterial lung infection	**Severe:**<br>*Actinobacteria*, *Firmicutes*<br><br>*Streptococcus*, *Actinomyces*, *Rothia*, *Eubacterium*, *Oribacterium*, *Mogibacterium*
Lee et al. 2019 [24]	IAV, IBV patients, Healthy controls	NP swab, OP swab	16S rRNA V4	**Genus**:<br>*Neisseria*	-	144	Household contacts = 573	Influenza cases = 124<br>Without viral shedding = 53<br>With viral shedding = 71		**Severe:**<br>Earlier signs of infection, longer symptom duration, longer viral shedding, shorter serial interval	**Severe:**<br>*Neisseria*
Ramos-Sevillano et al. 2019 [22]	IAV patients, Healthy controls	OP swab	16S rRNA V1-V3	**Genus**:<br>*Prevotella*	-	43	Healthy controls = 35	43	-	Symptomatic without complications	**Mild-moderate:**<br>*Prevotella*
Kaul et al. 2020 [20]	IAV patients, Healthy controls	NP swab	16S rRNA V1-V3	**Genus**:<br>*Pseudomonas*	**Phylum**:<br>*Actinobacteria*	28	Healthy controls = 22	28	-	Influenza-like illness	*Gammaproteobacteria*<br><br>*Pseudomonas*
Tsang et al. 2020 [26]	IAV, IBV patients, Household contacts	NP swab, OP swab	16S rRNA V4	-	-	IAV = 76<br>IBV = 39	Household contacts = 436	115	-	Symptomatic without complications	**Higher susceptibility to IBV:**<br>*Prevotella*<br><br>**Lower susceptibility to IAV, IBV:**<br>*Streptococcus*

Rattanaburi et al. 2022 [25]	IAV, IBV patients, COVID-19 patients, non-influenza and non-COVID-19 patients	NP swab	16S rRNA V4	**Family**: *Enterobacteriaceae*	**Phylum**: *Firmicutes*, *Bacteroidetes*, *Proteobacteria* **Genus**: *Streptococcus*, *Prevotella*, *Fusobacterium* **Species**: *Veillonella*	IAV = 24<br>IBV = 24	COVID-19 = 24<br>Non-influenza and non-COVID-19 = 24	48	-	Symptomatic without complications	**Mild-moderate:** *Enterobacteriaceae*
Borges et al., 2018 [27]	IAV with severe acute respiratory infection; Non-IAV with severe acute respiratory infection	NP aspirate	16S rRNA V4	-	-	6	Non-IAV = 6	-	6	Severe acute respiratory infection	**Severe:** *Firmicutes* *Streptococcus*
Hernández-Terán et al. 2021 [9]	IAV H1N1 patients with acute respiratory distress syndrome	BAL	16S rRNA V3-V4	-	-	30	-	-	Recovered = 25<br>Deceased = 5	**Severe:** Acute respiratory distress syndrome	**Recovered:** *Bacteroidetes*, *Actinobacteria*<br>**Deceased:** *Firmicutes*, *Proteobacteria*, *Granulicatella*

IAV: Influenza A virus; IBV: Influenza B virus; MP: *Mycoplasma pneumoniae*; NP: Nasopharyngeal; OP: Oropharyngeal; BAL: bronchoalveolar lavage
